# Minimum volume standards in day surgery: a systematic review

**DOI:** 10.1186/s12913-020-05724-2

**Published:** 2020-09-18

**Authors:** Michal Stanak, Christoph Strohmaier

**Affiliations:** 1 Austrian Institute for Health Technology Assessment (former Ludwig Boltzmann Institute for Health Technology Assessment), Vienna, Austria; 2 Department of Philosophy, University of Vienna, Vienna, Austria

## Abstract

**Background:**

The aim was to find out if and for what indications are minimum volume standards (MVS) applied in the day surgery setting and whether the application of MVS improves patient relevant outcomes.

**Methods:**

We conducted a comprehensive systematic literature search in seven databases on July 12th, 2019. Concerning effectiveness and safety, the data retrieved from the selected studies were systematically extracted into data-extraction tables. Two independent researchers (MS, CS) systematically assessed the quality of evidence using the quality assessment tool for individual studies of the International Society for Pharmacoeconomics and Outcomes Research (ISPOR) called Task Force Checklist for Quality Assessment of Retrospective Database Studies. No instances of disagreement occurred. No further data processing was applied.

**Results:**

The systematic literature search, together with hand search, yielded 595 hits. No prospective or controlled studies were found. Data from eight retrospective studies were used in the analysis of clinical effectiveness and safety on seven indications: anterior cruciate ligament reconstruction, cataract surgery, meniscectomy, thyroidectomy, primary hip arthroscopy, open carpal tunnel release, and rotator cuff repair. All interventions (except for carpal tunnel release and thyroidectomy) confirmed a volume-outcome relationship (VOR) with relation to surgeon/hospital volume, however, none established MVS for the respective interventions. Safety related data were reported without its relationship to surgeon/hospital volume.

**Conclusions:**

This present paper provides some evidence in favor of the VOR, however, it based on low quality retrospective data-analyses. The present results cannot offer any clear-cut MVS thresholds for the day surgery setting and so the simple transition from inpatient results (that support MVS) to the day surgery setting is questionable. Further quality assuring policy approaches should be considered.

## Background

### Definition of day surgery

The International Association for Ambulatory Surgery (IAAS) defines *day surgery* as a practice where patients are admitted, operated on, and discharged during the time frame of one working day (6 to 8 h), with no overnight stay [1]. The EU observatory as well as the British Association for Day Surgery (BADS) further add that *true* day surgery includes planned non-emergency surgical procedures on carefully-selected and prepared patients that are intended to be treated in the day surgery setting [2, 3]. Because some procedures may require longer recovery or observation, in order to keep them as day surgeries, they have to be performed in the morning sessions [3]. The US ambulatory care setting shares the definition of IAAS [4], yet at times, the US’ use of the term day surgery includes 23 h stay surgery, which in the EU is seen as an inpatient surgery with a 1 day length of stay (LOS). Alternative terms conveying the same meaning in different contexts are *same day surgery, ambulatory surgery, outpatient surgery, day-care hospital, day only*, or *zero day hospital stay*.

### Reasons for shifting to day surgery

Reasons for the shift from inpatient surgery to the day surgery context are manifold: organizational, ethical, economic, and medical. The aim is to improve the pathway of care, while alleviating the distributive justice mechanism and thus saving economic resources that can be allocated elsewhere. Day surgeries allow patients to choose their own surrounding to convalesce and they are associated with shorter waiting times and lower risks of surgery cancellation [5]. Also, day surgeries claim to reduce the rates of hospital-acquired infection and venous thromboembolism [5]. Furthermore, the consequences of shifting to day surgery seem to be a win-win situation for all parties involved [5]. Omitting overnight and weekend stays can be beneficial for the patient as well as for the medical team that does not have to stay at work over those times. This, in turn, saves the resources to the payers, potentially making the surgical interventions less expensive overall. In a report published by the UK NHS in 1989, costs of day surgery were estimated to be significantly lower compared to in-patient treatment [6]. In 2015, the Kings Fund verified the initial estimates stating that increases in day surgery over the period from 1998 to 2013 have generated savings of around £ 2 billion (€ 2.32 billion) [7]. Further patient-relevant consequences of day surgery are increase of control over patients’ own time and health as being called to come right before the intervention and then being followed by a telephone call after the intervention allows the patients to be more in charge [5]. Day surgery also potentially reduces the stigma that is associated with hospital admission as the patients return home within one working day.

### Current management in day surgery

According to the 2019 guideline from the Association of Anesthetists (AA) and the BADS, it is thanks to the advances in surgical and anesthetic techniques that even patients with multiple comorbidities can be treated in the day care setting. Hence, AA and BADS suggest a paradigm shift towards day surgery – meaning that if inpatient surgery is being considered, it is important to question whether any strategies could be employed to enable the patient to be treated as a day case [8].

They further state that three main selection criteria need to be considered when assessing patients’ eligibility: social, medical, and surgical [8]. Social criteria include the need of a responsible adult to escort the patient home and the presence of a carer for a required number of hours postoperatively in patient’s home setting. Medical criteria include patients’ fitness for the procedure and presence of a chronic disease. And surgical criteria require that the intervention does not carry a significant risk of serious postoperative complication that would need immediate medical attention. Postoperative symptoms must be controllable and patients should be able to mobilize before discharge. Treating a patient as a day case, hence, requires a well-organized day surgery unit that can communicate well with the patient before, during, as well as after the intervention. It is needed because patients should be admitted to the day surgery unit as close as possible to the time of their surgery and followed-up after the surgery once in their own home setting [8]. Patients should be provided with general, as well as procedure-specific, information that should be given to them in advance of admission and verbal comments should be reinforced with written material [8].

In terms of premises, day surgeries can be conducted in hospital outpatient departments, freestanding ambulatory surgery centers, or in office-based surgeries [4]. Also, the same hospital beds used for inpatient surgeries can be used for day surgery procedures. Concerning equipment and supplies, the same is needed for conducting inpatient as well as day surgery interventions, except for the presence of overnight beds.

### Theory of minimum volume standards

The theory of minimum volume standards (MVS) is based on the assumption that *practice makes perfect*. The assumption is that there is a relationship between volume and outcome, meaning that physicians, non-physician staff, or hospitals improve their (surgical) capabilities and outcomes with increasing volume of patients through a learning effect. The expected causality is that quantity affects quality. On the basis of available inpatient data on the volume-outcome relationship (VOR), health care decision-makers promote the implementation of regulatory MVS frameworks that should assure that surgeons or hospitals that comply with MVSs deliver their services at a certain level of quality. In most cases, annual volumes per surgeon or hospital are applied. A possible consequence of not complying with MVS is that surgeries are not reimbursed anymore [9].

In the absence of systematic reviews on MVS in the day surgery setting, we were commissioned by the Main Association of Austrian Social Security Institutions to conduct a systematic review on the topic. Our aim was to find out if and for what indications are MVS applied in the day surgery setting and whether the application of MVS improves patient relevant outcomes. For our review, we used the European Network for Health Technology Assessment (EUnetHTA) Core Model® for Rapid Relative Effectiveness Assessment (REA) and we adjusted the assessment elements to give us answers on the impact of MVS on clinical outcomes.

## Methods

### Systematic literature search and study selection

We conducted a comprehensive systematic literature search on July 12, 2019 in MEDLINE via OVID, Embase, the Cochrane Library, CRD (DARE, NHS-EED, HTA), and Livivo. The search was limited to years 2000 to 2019 and to articles published in English or German. The inclusion criteria for literature selection were defined using Population-Intervention-Comparison-Outcome-(Study design) model shown in Table 1. While no minimum number on patient population was applied, individual case reports were excluded. We did not search for specific interventions, but for any interventions that provided information on the VOR, or more specifically on the MVS. The target patient population was hence identified by the interventions with VOR/MVS.

**Table 1:** PICO inclusion criteria

**P**opulation	Patients suitable for day surgery, for example: β according to anesthesia risk classes (ÖGARI), β according to the type of anesthetic options (local, mask), β according to ASA classification or general illness/condition. The appropriate patients are identified in the literature analysis for the specific interventions/indications found. Key words: day surgery, same day surgery, ambulatory surgery, outpatient surgery, day-care hospital, day only, zero-day hospital stays
**I**ntervention	Identified surgical interventions from the international literature in the day surgery setting that implement minimum volume standards.
**C**ontrol	The same or comparable surgical interventions in the day surgery setting without minimum volume standards implemented.
**O**utcomes	Depending on the identified indications/interventions, general health-relevant results such as morbidity, mortality, functional outcomes such as functionality in everyday life or at the workplace, quality of life or satisfaction are taken into account. In addition, results are specifically considered for outpatient interventions such as frequency of hospital infections and venous thrombosis embolisms, etc.
Setting	Day surgery/outpatient care/day clinic/zero-day stays/same-day surgery
Study design	No limitation
Publication period	2000–2019
Language	English/German

### Analysis and synthesis

For the systematic literature search on effectiveness and safety, the data retrieved from the selected studies were systematically extracted into data-extraction tables (see Tables 2 and 3). No further data processing such as indirect comparison was applied. Two independent researchers (MS, CS) systematically assessed the quality of evidence using the quality assessment tool for individual studies of the International Society for Pharmacoeconomics and Outcomes Research (ISPOR) called Task Force Checklist for Quality Assessment of Retrospective Database Studies [18]. No instances of disagreement occurred. Due to the retrospective study design of the evidence base, data on each selected outcome category were not synthesized across studies according to Grading of Recommendations Assessment, Development and Evaluation (GRADE).

**Table 2:** from retrospective database analyses (joints and carpal tunnel)

	Degen et al. [10] (2017)	Evers et al. [11] (2018)	Jain et al. [12] (2005)	Jain et al. [13] (2004)	Liu et al. [14] (2018)
**Country**	USA	The Netherlands	USA	USA	USA
**Sponsor**	National Institute of Health/ National Institute for Arthritis and Musculoskeletal and Skin Diseases (R01AR066069)	National Institute of Health/ National Institute of Arthritis and Musculoskeletal and Skin Diseases (RO1 AR82813)	NA	NA	NA
**COI**	1 author (B.T.K) has COI due to fees form Arthrex and A-3 surgical	None	NA	NA	None
**Study design**	Retrospective database analysis of 137 surgical centres (multivariate regression)	Retrospective database analysis of 11 surgical centres (univariate and multivariate regression)	Retrospective database analysis of unclear number of surgical centres (univariate and multivariate regression)	Retrospective database analysis of unclear number of surgical centres (multivariate logistic and linear regression)	Retrospective database analysis of unclear number of surgical centres (multivariate regression)
**Conducted in**	1998–2012	2011–2015	1997–2000	1997–2000	2009–2013
**Indication**	Hip arthroscopy	Carpal tunnel syndrome	See inclusion criteria below	See inclusion criteria below	Anterior cruciate ligament injury
**Intervention**	Primary hip arthroscopy	Open carpal tunnel releases	Rotator cuff repair	ACL reconstruction & Meniscectomy	ACL reconstruction
**Setting**	Outpatient	Outpatient	Outpatient	Outpatient	Outpatient
**Type of volume analysis (surgeon/hospital)**	Surgeon	Surgeon	Surgeon & hospital	Surgeon & hospital	Hospital
**Comparator**	NA	NA	NA	NA	NA
**Number of pts**	7836	2057^a^	10,934^b^	*ACL:* 32,440 *Men:* 195,597^c^	26,873
**Number of procedures**	8267^d^	2057	NA	NA	NA
**Number of surgeons**	295	17	NA	NA	NA
**Number of hospitals**	137	11	NA	NA	NA
***Surgeon volume categories, n (cases/year)***				*ACL/Men:*	
β Low	< 102	6–44	< 15^e^	< 25^f^/< 75	NA
β Medium	102 ≤ x < 164	47–71	15 ≤ x < 30	25 ≤ x < 75/75 ≤ x < 175	
β High	164 ≤ x < 340	75–163	≥30	≥75/≥175	
β Very high	≥340	NA	NA	NA	
***Hospital volume categories, n (cases/year)***				*ACL/Men:*	
β Low	NA	NA	< 75	< 125/< 600^g^	< 1006
β Medium	NA	NA	≥75- < 200	125 ≤ x < 300/600 ≤ x < 1200	≥100- < 500
β High	NA	NA	≥200	≥300/≥1200	≥500
**Operating time, median in min (range)**	NA	NA	102 (30–595)^h^	*ACL:* 125 (NA) *Men:* 55 (NA)	NA
**Inclusion criteria**	HA for diagnosis with or without synoval biopsy, HA for removal of loose/foreign body, HR & chondroplasty, abrasion arthroplasty & resection of labrum, HA & synovectomy, HA with femoroplasty, HA with acetabuloplasty, HA with labral repair, total hip replacement, resurfacing hip & partial/total acetabulum & femoral head	Consent, primary carpal tunnel release, baseline and follow-up measurement of BCTQ	Rupture of the rotator cuff, disorders of bursae and tendon, sprains and strains of the rotator cuff capsule	*ACL:* Complete rupture of old disruption of ACL and sprain of cruciate ligament of the knee *Men:* derangement, bucket handle tear, simple tear of the meniscus or cartilage	NA
**Exclusion criteria**	NA	Unavailable operative report, unidentified surgeon, cases in which surgeons did not perform CTRs for at least 1 year within the cohort	Shoulder bone infection, present surgery as corrective surgery, malignancy, pathologic fracture, fracture due to injury in the bones of the shoulder region, or simultaneous total or partial shoulder arthroplasty	*ACL/Men:* Lower leg bone infections like osteomyelitis, inflammatory reaction due to graft, correction surgery, malignancies or pathological fractures, fractures due to injury, simultaneous knee arthroplasty, rheumatoid arthritis, operating time < 45 min & < 20 min in case of meniscectomy	Non-New York residents, pts. that left against medical advice, mortalities, pts. < 18 yrs., surgeries from December 2013
**Co-interventions**	NA	Trigger finger release, cubital tunnel relese, Guyon release, radial tunnel release, fasciotomy Dupuytren, other, standard postoperative care – nerve and tendon-gliding exercises	NA	NA	NA
**Age, mean, yrs (range)**	38 (7–84)	54 (41–67)	56 (43.6–68.4)	*ACL:* 29.4 (18.9–39.9) *Men:* 47.3 (31.9–62.7)	Average 33.3 (22.0–46.6)
**Sex, female:male, n**	4443:3801	986:359	3785-6188	*ACL:* 7481: 10,908^i^ *Men:* 50,108:72,889^j^	9811:17,049
**BMI ± SD**	NA	27 ± 5	NA	NA	NA
**Comorbidities**	NA	Diabetes mellitus, Rheumatoid arthritis, Dupuytren’s disease, Trigger fingers, CMCI joint arthritis, compression neuropathy, tendinitis, history of wrist trauma, scaphotrapeziotrapezoidal joint arthritis, radiocarpal arthritis, peripheral neuropathy, cervical radioculopathy, ulnocarpal impingement	Mean Deyo score: 0.1–0.9	NA	Mean Deyo score: 0.06
**Follow-up time, yrs**	10^k^	0.5	NA	NA	30 days
**Patients excluded from the analysis, n (%)**	?	712	961	*ACL:* 14,050 *Men:* 72,585	NA
**Efficacy**					
**Survival, %**	At 2/5/10 yrs				
β Low	86.5/78.5/72.7	NA	NA	NA	NA
β Medium	87.8/82.7/82.7				
β High	94.6/90.2/90.2				
β Very high	97.6/97.6/97.6				
**Reduction in pain score units on VAS scale, baseline/6 mo by volume, mean n (SD)**					
β Low	NA	Low: 47/18^l^	NA	NA	NA
β Medium		Medium: 51/20			
β High		High: 51/19			
β *P* value		NA			
**BCTQ, mean n**					
β Symptom severity score	NA	Low&Medium&High: all 1.7^m^	NA	NA	NA
β Functional status score	NA	Low&Medium&High: all 1.7	NA	NA	NA
**Hospital based acute care within 30 days, odds ratio n at 5 years (p value)**					
β Low volume	NA	NA	NA	NA	x
β Medium volume	NA	NA	NA	NA	0.77 (p 0.059)
β High volume	NA	NA	NA	NA	0.47 (*p* < 0.001)
**Risk of reoperation, hazard ratio n (95% CI)**					
β Low volume	x				
β Medium volume	0.9x (CI-0.74)	NA	NA	NA	NA
β High volume	0.42 (CI 0.32)	NA	NA	NA	NA
β Very high volume	0.17 (CI 0.07)	NA	NA	NA	NA
**Nonroutine disposition of pts at discharge**^n^**, surgeon, n (95% CI)**					
β Low volume	NA	NA	2.8x (0.9–9.1)	*ACL:* 0.9% *Men:* 1.4%	NA
β Medium volume	NA	NA	1.5x (0.7–3.1)	*ACL:* 0.4% *Men:* 0.7%	NA
β High volume	NA	NA	x	*ACL:* 0.2% *Men:* 0.5%	NA
**Nonroutine disposition of pts at discharge, hospital, n (95% CI)**					
β Low volume	NA	NA	2.1x (0.6–8.0)	*ACL*: 1% *Men:* 1.6%	NA
β Medium volume	NA	NA	1.7x (0.2–11.6)	*ACL:* 0.2% *Men:* 1.2%	NA
β High volume	NA	NA	x	*ACL:* 0.3% *Men:* 0.2%	NA
**Mean operating time volume, surgeon, min (±SD)**					
β Low volume	NA	NA	112 (4)	*ACL:* 149(9) *Men:* 72(6)^o^	NA
β Medium volume	NA	NA	113 (4)	*ACL:* 137(9) *Men:* 64(6)	NA
β High volume	NA	NA	102 (4)	*ACL:* 122(9) *Men:* 53(6)	NA
β p value	NA	NA	< 0.001	NA	NA
**Mean operating time volume, hospital, min (±SD)**					
β Low volume	NA	NA	111	*ACL:* 150(9) *Men:* 71(5)15	NA
β Medium volume	NA	NA	109	*ACL:* 132(9) *Men:* 66(6)	NA
β High volume	NA	NA	105	*ACL:* 129(14) *Men:* 52(6)	NA
**LOS and surgeon volume, n (95% CI)**					
β Low volume	NA	NA	2.3x (1.2–4.4)	NA	NA
β Medium volume	NA	NA	1.3x (0.7–2.6)	NA	NA
β High volume	NA	NA	x	NA	NA
**LOS and hospital volume, n (95% CI)**					
β Low volume	NA	NA	0.5x (0.2–1.1)	NA	NA
β Medium volume	NA	NA	1.1x (0.4–3.1)	NA	NA
β High volume	NA	NA	x	NA	NA
**Safety**					
**SAE**	NA	NA	NA	NA	NA
**AEs, n (%)**	NA (0.2)^p^	23 (1.6)	NA	NA	Unclear^q^
β Day surgery related AEs	MI, ileus, pneumonia, sepsis, mechanical complication, hardware failure, DVT/PE, wound infection, dislocation/iatrogenic instability, major bleed^r^	Wound infection, wound dehiscence	NA	NA	NA

*ACL* Anterior cruciate ligament, *BCTQ* Boston Carpal Tunnel Questionnaire, *COI* Conflict of interests, *CTR* Carpal tunnel release, *DVT* Deep vein thrombosis, *ED* Emergency department, *HA* Hip arthroscopy, *LOS* Length of stay, *THA* Total hip arthroplasty, *Men* Meniscectomy, *MI* Myocardial infarction, *NA* Not available, *PE* Pulmonary embolism

^a^1345 pts. included in the analysis

^b^Number of pts. finally included in the analysis was 9973. Exclusion criteria were applied to exclude diagnoses, which outcomes were expected to differ from outcomes of the included indications

^c^Number of pts. finally included in the analysis was 18,390 for ACL and 123,012 for meniscectomy

^d^Including 23 simultaneous bilateral and 408 staged bilateral procedures

^e^Number of cases in the period of 1997–2000

^f^Number of cases per 4 years

^g^Number of cases per 5 years

^h^Data missing on 123 pts. (0.01%)

^i^Records missing on 1 pt

^j^Records missing on 15 pts

^k^Analysis of volume/risk of reoperation relationship assessed at 5 years

^l^The scores were reported only in a table and so the following numbers are only authors’ estimates

^m^The scores were reported only in a graph and so the following numbers are only authors’ estimates

^n^Nonroutine disposition included transfer to another hospital, skilled nursing facility, intermediate care facility, or home health care. Routine disposition reflected patients who were discharged home

^o^Only restricted to the New York state database pts

^p^Thirty days procedural complication rate

^q^Listed complication are not necessarily intervention related, they are merely the reasons due to which pts. visited EDs within 30 days of ACL surgery

^r^Not reported in what n of pts., nor in relationship to surgeon volume

**Table 3:** Results from retrospective database analyses (thyroid and cataract surgery)

	Ayala and Yencha [15] (2015)	Chen et al. [16] (2014)	Keay et al. [17] (2012)
**Country**	USA	USA	USA
**Sponsor**	NA	NA	National Eye Institute: R01EY016769. K.K funded by an Australian National Health and Medical Research Council post-doctoral fellowship. E.W.G. recipient of an Ernest and Elizabeth Althouse Special Scholar’s Award from Research to Prevent Blindness.
**Conflict of Interest**	One author (Yencha) was involved in all cases either as primary or assistant surgeon	None	None
**Study design**	Retrospective single centre analysis	Retrospective single centre chart review	Retrospective analysis of Medicare beneficiary claims data
**Conducted in**	2006–2014	2011–2012	2003–2004
**Indication**	Benign or malignant thyroid carcinoma	Cataract	Cataract
**Intervention**	Outpatient thyroid surgery/Thyroidectomy	Cataract Surgery (Phacoemulsification)	Cataract Surgery
**Setting**	Outpatient and Inpatient^a^	Outpatient Surgical Centre	Outpatient surgery centres
**Type of volume analysis (surgeon/hospital)**	Surgeon & Hospital	Surgeon	Surgeon
**Comparator**	Inpatient Thyroid surgery/Thyroidectomy	NA	NA
**Number of pts, I vs C**	160 (109 vs 51)^b^	3339	2,285,968^c^ Both eyes: 1,005,826 (44%) One eye: 1,280,14221 (56%)
**Number of procedures, I vs C**^d^	Total: 35 vs 26 Hemi: 62 vs 20 Completion: 11 vs 5	NA	3,280,966^e^
**Number of surgeons**	NA	4	11, 873^f^
**Number of hospitals**	1	1	NA
**Surgeon/Surgeon volume categories, n (cases/year)**	Unclear^g^	Surgeon 4: 411 Surgeon 1: 536 Surgeon 2: 1056 Surgeon 3: 1336	(1): 1–50; (2): 51–200; (3): 201–500; (4): 501–1000; (5): ≥1001
**Hospital volume categories, n (cases/year)**	Unclear	NA	NA
**Operating time, median in min (range)**	NA	NA	NA
**Inclusion criteria**	Patients in ASA class 1,2,3 and 4	Use of topical anaesthesia and performance of the intervention at an outpatient centre/setting	Patients with max. 2 cataract surgeries per beneficiary during the 2-year study timeframe; Medicare beneficiaries ≥65 years
**Exclusion criteria**	NA	Patients requiring additional anaesthesia and those who were operated on in a hospital setting	Records were excluded if data indicated the surgery was not performed, the procedure was a return to the operating room for a related procedure or due to data coding issues; surgeries performed in the last 42 days of 2004
**ASA class, n, I vs C**	1 and 2: 90 vs 39 3 and 4: 19 vs 12	NA	NA
**Co-interventions**	Intravenous dexamethasone, intravenous antibiotics, anaesthesia at surgeon’s discretion, Prophylactic calcium carbonate and vitamin D (calcitriol) supplementation for pts. undergoing total or completion thyroidectomy	NA	NA
**Age, mean, yrs (range) [SD]**	41.8 (14–75)/47.8 (19–77)	73 (60–86) [3]	NA (≥65^h^)
**Sex, female:male, n, I vs C**	82:27 vs 25:26	13:10	NA
**BMI ± SD**	NA	NA	NA
**Risk Adjustment**	NA	NA	Age (65–74, 75–84, ≥85 Gender, Race, Year, Ambulatory surgery centre (No, Yes), Surgeon experience in yrs. (1–10, 11–20, 21–30, ≥30)
**Other influencing factors** **(Comorbidities etc.), n (%)**	NA	Shallow chamber: 8 (35); Miosis: 7 (30); Restlessness: 6 (26); Floppy Iris: 6 (26); Pseudoexfoliation: 5 (22); Zonular dehiscence: 5 (22); Small eye: 1 (4)	NA
**Patients excluded from the analysis, n (%)**	NA	NA	165,452 and 35,068^i^
**Efficacy**			
**Revision Surgery**	NA	23^j^ of 3339	NA
**Re-admission**^k^**, n (%), I vs C**	0^l^ vs NA	NA	NA
**Surgical volume,** **n (cases/year) – Risk of AE related** **to surgery**	NA	411–3.75; 536–0.37; 1056–0.28; 1336–0.29 PCR in 23 (0.68) in total	Nr. of TE cases: 1–50: 352; 51–200: 1455; 201–500: 1512; 501–1000: 454; ≥1001: 168 Overall Endoph. Rate/1000 surgeries^m^ (95% CI): 1–50: 2.57 (2.30–2.83); 51–200: 1.49 (1.42–1.57); 201–500: 1.17 (1.11–1.23); 501–1000: 0.80 (0.73–0.88); ≥1001: 0.62 (0.52–0.71)
**Surgical volume,** **n (cases/year) – Risk of AE related** **to surgery** *(continuation)*			PCR: Unadjusted RR (95% CI): 1–50: 4.17 (3.47–5.01); 51–200: 2.42 (2.06–2.84); 201–500: 1.89 (1.61–2.22); 501–1000: 1.30 (1.09–1.55); ≥1001: 1.00 (Reference) Adjusted RR: (95% CI): 1–50: 3.80 (3.13–4.61); 51–200: 2.32 (1.97–2.74); 201–500: 1.84 (1.56–2.17); 501–1000: 1.30 (1.09–1.56); ≥1001: 1.00 (Reference)
**Safety**			
**SAE**	NA	NA	NA
**AEs, Volume, n** **(%),**	19 pts. of 160 pts. (11,90%)^n^ Transient hypercalcemia: 5%; Temporary vocal cord paralysis: 2.5%; Bilateral vocal cord paralysis: 0.63% Inadvertent transection of the RLN: 0.63%; Post-operative seromas requiring aspiration: 1.9%; Post-operative hematoma requiring aspiration: 1.25%	NA	NA

*NA* Not available, *PCR* Post Cataract Endophthalmitis, *SDS* Same day surgery, *SDT* Same-day thyroidectomy, *TE* Total Endophthalmitis, *TT* Thyroidectomy, *RLN* Recurrent laryngeal nerve, *RR* Relative Risk

^a^Patient who were eligible for same day discharge were observed typically for 2–4 h. Patients with significant co-morbidities, lack of social support, and/or patients not comfortable with outpatient recovery were admitted for observation

^b^Forty-three points were kept for 23 h observation and 17 (40%) of these patients were found to have social factors requiring an overnight stay (due to long distance, absence of responsible adult caregiver); remaining 26 pts. requiring a 23 h observation had significant co-morbidities

^c^Own calculation on the basis of the given numbers of patients (absolute and relative) with surgery on both eyes.

^d^Outpatient (Intervention) vs Inpatient (Comparator)

^e^35,068 surgeries could not have been attributed to a specific surgeon and also contain surgeries for which surgeon characteristics data were missing. Hence in the analysis 3,245,898 were included.

^f^Own calculation on the basis of the descriptive statistics of the endophthalmitis rate by annual Medicare surgical volume found in Table 4.

^g^Thresholds for MVS classification (i.e. low, medium, high) is not clear

^h^Age info is not given in detail

^i^165,452 Surgeries performed in the last 42 days in 2004 were excluded and in the analysis of the endophthalmitis rate by annual medicare surgical volume 35,068 surgeries with unique physician identification numbers that cannot be attributed to a specific surgeon and surgeries for which surgeon characteristics data were missing

^j^Five of the 23 patients did not have sufficient support to place a posterior chamber or sulcus intraocular cataract lenses (IOL) and required placement of anterior chamber IOLs

^k^Re-admission includes admission for 23-h observation or full admission (observation longer than 24 h)

^l^One pt. was discharged from the ER for symptoms of paresthesias with normal calcium levels

^m^This rate is overall for all surgeries within a specific annual volume category and does not reflect the average rate of endophthalmitis within each category

^n^It was unclear what AE occurred in the respective intervention arm

Outcome measures were specified once interventions with VOR/MVS were found. Crucial outcomes were selected according to EUnetHTA guidelines [19, 20] that suggest using clinical outcomes relevant for patients (mortality, morbidity, health-related quality of life, and satisfaction) and not surrogate end points. The eight studies included reported on seven different indications: primary hip arthroscopy, carpal tunnel release, rotator cuff repair, ACL reconstruction, meniscectomy, thyroidectomy, and cataract surgery. The description of intervention and the outcomes assessed in relation to volume (surgical/hospital) are listed below in Table 4.

**Table 4:** List of outcomes used in the assessment

*Intervention*	Thyroid surgery (thyroidectomy)	Cataract surgery (phacoemulsification)	Hip arthroscopy	Carpal tunnel release (decompression surgery)	Rotator cuff repair	ACL reconstruction	Meniscectomy
*Intervention description*	Removal of part or all of the thyroid gland	Removal of the lens of the eye and replacement it with an artificial lens	Surgical procedure used for diagnosis as well as treatment of hip related disorders	Surgical treatment of the carpal tunnel syndrome –compressed nerves in the wrist	Surgical treatment of rotator cuff disorders -inflamed or irritated shoulder tendons	Surgical treatment of ACL injuries -tears or sprains	Surgical treatment of meniscal injuries
*Volume perspective*	Surgeon	Surgeon	Surgeon	Surgeon	Surgeon/Hospital	Surgeon/Hospital	Surgeon/Hospital
*Outcomes*	- Admission for 23 h observation - Full admission (longer than 23 h) - AEs	- Risk of revision surgery^a^ - Risk-adjusted incidence of post-surgery endophthalmitis	- Risk of reoperation^a^ - Survival rates at two, five, and 10 yrs.^a^ - AEs	- BCTQ score^b^ was measured at 6 weeks, 3 months, and 6 months full admission (longer than 23 h)^a^ - Reduction in pain score units on VAS^c^ measured 6 weeks, 3 months, and 6 months - AEs	- Non-routine disposition of patients at discharge^da^ - LOS^ea^ - Mean operating time^f^	- Non-routine disposition of patients at discharge^a^ - Inpatient hospital admissions or emergency department visits within 30 days of surgery^ga^ - Mean operating time	- Non-routine disposition of patients at discharge^a^ - Mean operating time

*Abbreviations*: *ACL* Anterior cruciate ligament, *AEs* Adverse events, *BCTQ* Boston Carpal Tunnel Syndrome Questionnaire, *hrs* hours, *LOS* Length of stay, *VAS* Visual analogue scale, *yrs.* years

^a^refers to those outcomes that we considered crucial

^b^Two domains of BCTQ were assessed in particular: the Symptom Severity Scale (11 items) and the Functional Status Scale (8 items). Each item consists of five response categories ranging from one to five, where higher score represents worse symptoms/lower level of function

^c^VAS ranged from 0 to 100

^d^Non-routine disposition includes transfer to another hospital, skilled nursing facility, intermediate care facility, or home health care (health care provided at home by licensed health professionals)

^e^LOS was divided into two categories: less than 1 day and greater than or equal to 1 day – that was termed extended length of stay

^f^Operating room time was calculated in minutes for every procedure and defined as the total time spent in the operating room exclusive of preoperative and postoperative time

^g^Assessed from hospital perspective only

## Results

Overall, 538 citations were included after deduplication and the specific search strategies can be found in the supplementary material. Additional 57 citations we found via hand search, which resulted in overall 595 hits. No prospective or controlled studies were found by the systematic literature search and one of the studies had a control group [21]. Data from eight retrospective studies (and seven indications) were used in the analysis of clinical effectiveness and safety. Two studies were found on the intervention of anterior cruciate ligament (ACL) reconstruction [22, 23], two on cataract surgery [24, 25], and one for each of the following interventions: meniscectomy, thyroidectomy, primary hip arthroscopy, open carpal tunnel release, and rotator cuff repair [21, 26–28].

### Study characteristics

While two studies were single center analyses [21, 25], six studies were analyses of health care databases [22–24, 26–28]. Seven studies were conducted in the US [21–26, 28] and the eighth study was conducted in the Netherlands [27]. Information about study sponsors was not disclosed in five studies [21–23, 25, 26], two studies were funded by the National Institutes of Health/National Institute for Arthritis and Musculoskeletal and Skin Diseases [27, 28], and one study was funded by the National Eye Institute [24]. Two studies did not report on conflict of interests (COI) [23, 26], four studies reported that none of the authors had COI [22, 24, 25, 27], and two studies reported COI of one of their authors [21, 28]. The dates of data collection in all the studies were between 1997 and 2015.

All the studies gathered data on the outpatient setting and while four studies analyzed the VOR from the perspective of surgeons [24, 25, 27, 28], one analyzed it from the hospital perspective [22], and three from the perspectives of both surgeons as well as hospitals [21, 23, 26]. Follow-up time was not reported in five studies [21, 23–26], it was 10 years in [28], 6 months in [27], and 30 days in [22].

### Patient characteristics

The analysis of primary hip arthroscopy included 7836 patients and 8267 procedures that were performed by 295 surgeons in 137 centers [28]. The analysis of carpal tunnel release included 1345 patients/procedures (712 patients not followed-up) performed by 17 surgeons in 11 centers [27]. The analysis of rotator cuff repair included 9973 patients (961 not followed-up) [26], ACL reconstruction included 45,262 patients (14,050 not followed-up) [22, 23], and the analysis of meniscectomy included 123,012 patients (72,585 not followed-up) [23]. The number of procedures, surgeons, or surgical centers was not reported in the three studies above. For the single center analysis of thyroidectomy, 109 outpatient and 51 inpatient patients were included with 35 and 26 procedures respectively [21]. For the analysis of cataract surgery, 2,289,307 patients were included (200,520 not followed-up) with 3,280,966 procedures conducted by 22,877 surgeons in an unclear number of centers (except for four surgeons that were part of a single center analysis [25]). Because of the retrospective nature of the studies, loss to follow-up was not reported.

Surgeon as well as hospital volume was categorized into low, medium, and high (very high in one study [28]) and the thresholds differed with interventions. The low volume threshold ranged from six to 411 interventions, while the high (or very high) threshold ranged from 30 to 1336 interventions per year. Inclusion and exclusion criteria were heterogeneous as they varied with interventions. Co-interventions were reported in four studies [21, 22, 26, 27] and the mean age ranged from 29,4 to 73 years. Study characteristics and results of included studies are displayed in Tables 2 and 3.

### Clinical effectiveness

Concerning *cataract surgery*, inverse VOR was observed [24]. The surgeon volume rates ranged from 1 to 50 (1), 51–200 (2), 201–500 (3), 501–1000 (4) and ≥ 1001 (5). The number of cases per surgeon was inversely correlated with the adverse event of posterior capsule rupture (PCR), where PCR and vitreous loss (VL) rate were 3.75% for low volume and 0.29% for very high volume surgeons [25]. The relative risk (RR) for endophtalmitis was 4-fold between low and very high volume surgeons [24]. After adjusting for risk factors the RR in category (1) was still 3.8-fold higher than in the reference category (5).

Concerning *thyroidectomy*, there was no VOR observed, but it was suggested that thyroidectomy is safe also in low volume centers as in the single low volume center, no cases of readmission occurred [21]. The surgeon volume amounted to 10 thyroid surgical cases per year on average, while the hospital volume averaged of 20 thyroid surgical cases per year.

Concerning *rotator cuff repair*, VOR was observed. The surgeon volume rates ranged from low (< 15), medium (15–30), to high (≥30), while hospital volume rates ranged from low (< 75), medium (75–200), to high (≥200). Patients of low volume surgeons were 2.8 time more likely to have nonroutine disposition at discharge, while low volume hospitals were 2.1 times more likely to discharge patients with nonroutine dispositions. Surgeon-related mean operating time was 10 min shorter and hospital-related mean operating time was 6 min shorter for high volume compared to low volume surgeons/hospitals. Length of stay (LOS) was 2.3 times longer for low volume surgeons and 0.5 times for low volume hospitals compared to high volume surgeons/hospitals [26].

Concerning *hip arthroscopy*, VOR was observed. Surgeon yearly volume rates ranged from low (< 102), medium (102–164), high (164–340), to very high (≥340). The survival rates for very high volume surgeons were 11.1–24.9% higher than for low volume and the hazard ratio for reoperation (with reference value of low volume) was 0.17 for very high volume surgeons [28].

Concerning *open carpal tunnel release*, VOR was not observed. Surgeon yearly volume rates ranged from low (6–44), medium (47–71), to high (75–163). BCTQ score did not vary with volume at all while the difference on the VAS scale was 1 point (out of 100) between low and high volume surgeons (18 vs. 19 points) [27]. Such difference is below the threshold of the minimal clinically important difference [15].

Concerning *ACL reconstruction*, VOR was observed. The surgeon volume rates ranged from low (< 25), medium (25–75), to high (≥75) and hospital volume rates ranged from low (< 125), medium (125–300), to high (≥300). The odds ratio for hospital based acute care within 30 days (with reference of low volume hospitals) was 0.47 for high volume hospitals [22]. Low volume surgeons were 4.5 times more likely and low volume hospital 3.33 times more likely to have nonroutinely discharged patients compared to high volume surgeons [23]. Furthermore, low volume surgeons had a 27 min longer and low volume hospitals 21 min longer mean operating time than high volume surgeons/ hospitals [23].

Concerning *meniscectomy*, VOR was observed. The surgeon volume rates ranged from low (< 75), medium (75–175), to high (≥175) and the hospital volume rates ranged from low (< 600), medium (600–1200), to high (≥1200). The low volume surgeons were 2.8 times and low volume hospitals were 8 times more likely to have nonroutinely disposed patients at discharge than high volume surgeons/hospitals [23]. In terms of mean operating time, both low volume surgeons and low volume hospitals had a longer mean operating time by 19 min compared to high volume surgeons/hospitals [23].

### Safety

The only safety related data reported were without its relationship to surgeon/hospital volume. In the *hip arthroscopy* study, 0.2% of patients experienced procedural complication at 30 days post intervention – the complications were: myocardial infarction ileus, pneumonia, sepsis, mechanical complication, hardware failure, deep vein thrombosis/pulmonary embolism, wound infection, dislocation/iatrogenic instability, major bleed [28]. In the *carpal tunnel release* study, 1.6% of patients experienced procedural complications such as wound infections, wound dehiscence [27].

In the *thyroidectomy* study [21], 19 of the 160 patients experienced complications. Complications included transient hypercalcemia (5%), temporary vocal cord paralysis (2.5%), post-operative seromas requiring aspiration (1.9%), post-operative hematoma requiring aspiration (1.25%), bilateral vocal cord paralysis (0.63%), and inadvertent transection of the recurrent laryngeal nerve (0.63%).

## Discussion

### Clinical effectiveness and safety

To our knowledge, this is the first systematic review on minimum volume standards (MVSs) applied to the general setting of day surgery. The 2012 report of the German Institute for Quality and Efficiency in Healthcare (IQWiG) on effects of minimum volume regulations was set out to evaluate outpatient evidence only, however, it also included inpatient data [16].

### Summary of evidence from retrospective database analysis

Focusing exclusively on the day surgery setting, we found no prospective or controlled trials for the analyses of clinical effectiveness and safety. We found eight retrospective database analyses on seven different indications, but none reported on the volume-outcome relationship (VOR) with respect to safety. Each indication included the following number of patients:

- β thyroidectomy – 109 outpatient (and 51 inpatient) patients [21],
- β cataract surgery – 2,289,307 patients [24, 25]
- β primary hip arthroscopy – 7836 patients [28],
- β open carpal tunnel release – 1345 patients [27],
- β rotator cuff repair – 9973 patients [26]
- β ACL reconstruction – 45,262 patients [22, 23]
- β and meniscectomy – 123,012 patients [23].

All interventions (except for carpal tunnel release and thyroidectomy) confirmed the VOR, however, none established MVS for the respective interventions.

### Gaps in evidence

While the VOR has some standing in the inpatient setting [14], it is argued that that relationship is based on low quality of evidence [17]. That is even more true for the day surgery setting and because of the fact that day surgery centers may operate independently from hospitals and so miss on the safety net in the form of emergency departments, quality assurance in day surgery is of even more importance. That places the extra emphasis on day surgery interventions to go well in the first place and hence also on quality assurance measures such as MVSs.

### Relevance of evidence

Due to these gaps in evidence, the relevance of the current evidence base to relative effectiveness assessment of MVSs is questionable. Retrospective database analyses do not fulfil the evidence-based medicine standards as they are prone to a spectrum of biases. For that reason, their conclusions are applicable only in part. In their favor plays the relatively robust body of evidence from the inpatient setting, relatively high number of patients included in the day surgery studies, and studies supporting no significant difference in outcomes between the settings [2]. Against it plays the poor internal and external validity of the present evidence base and the critical considerations related to MVSs in general (outlined below).

### Internal and external validity of the present evidence base

Concerning effectiveness and safety of MVSs as assessed by the ISPOR checklist in Table 5, the quality of the evidence base is very low. The main reasons are the retrospective design of all the studies [21–28], the lack of justification for its use [21, 23, 25–27], or the lack of a priori data analysis plan [21–28]. Further reasons include unclear eligibility criteria [21, 22, 25, 27, 28], lack of justification for the statistical models used [21–23, 25–28], lack of interpretation of the statistical findings in terms of their clinical or economical evidence [22, 23, 25–28], and limited recognition of the generalizability of the retrospective study design [21, 23, 25–27].

**Table 5:** ISPOR task force checklist for quality assessment of retrospective database studies [29]

Study reference/ID	Degan et al. [10] (2017)	Evers et al. [11] (2018)	Jain et al. [12] (2005)	Jain et al. [13] (2004)	Liu et al. [14] (2018)	Ayala and Yencha [15] (2015)	Chen et al. [16] (2014)	Keay et al. [17] (2012)
1. Relevance: Have the data attributes been described in sufficient detail for decision makers to determine whether there was a good rationale for using the data source, the data source’s overall generalizability, and how the findings can be interpreted in the context of their own organization?	Yes	Yes	Yes	Yes	Yes	Yes	Yes	Yes
2. Reliability and Validity: Have the reliability and validity of the data been described, including any data quality checks and data cleaning procedures?	No	Yes	Yes	Yes	No	No	Yes	Yes
3. Linkages: Have the necessary linkages among data sources and/or different care sites been carried out appropriately, taking into account differences in coding and reporting across sources?	Yes	NA	Yes	Yes	Yes	NA^a^	NA^a^	Yes
4. Eligibility: Have the authors described the type of data used to determine member eligibility?	Yes	Yes	Yes	Yes	Yes	No	No	Yes
5. Data analysis plan: was a data analysis plan, including study hypotheses, developed a priori? Was the study conducted prospectively?	Partial^b^	Partial^b^	Partial^b^	Partial^b^	Partial^b^	No^c^	No	Partial^b^
6. Design selection: has the investigator provided a rationale for the particular research design?	No	No	No	No	No	No	No	Partial^d^
7. Research design limitations: did the author identify and address potential limitations of that design?	Yes	No	No	No	Yes	No	No	Partial
8. Treatment effect: for studies that are trying to make inferences about the effects of an intervention, does the study include a comparison group and have the authors described the process for identifying the comparison group and the characteristics of the comparison group as they relate to the intervention group?	NA	NA	NA	NA	NA	NA	NA	NA
9. Sample selection: have the inclusion and exclusion criteria and the steps used to derive the final sample from the initial population been described?	Partial^e^	Partial^f^	Yes	Yes	Partial^g^	No	Partial^f^	Yes
10. Eligibility: are subjects eligible for the time period over which measurement is occurring?	NA	NA	NA	NA	NA	NA	NA	NA
11. Censoring: were inclusion/exclusion or eligibility criteria used to address censoring and was the impact on study findings discussed?	Partial^h^	Partial^h^	Partial^h^	Partial^h^	Partial^h^	No	Partial^h^	Yes
12. Operational definitions: are case (subjects) and end point (outcomes) criteria explicitly defined using diagnosis, drug markers, procedure codes, and/or other criteria?	Yes	Yes	Yes	Yes	Yes	Partial^i^	Yes	Yes
13. Definition validity: have the authors provided a rationale and/or supporting literature for the definitions and criteria used and were sensitivity analyses performed for definitions or criteria that are controversial, uncertain, or novel?	NA	NA	NA	NA	NA	NA	NA	NA
14. Timing of outcome: is there a clear temporal (sequential) relationship between the exposure and outcome?	NA	NA	NA	NA	NA	NA	NA	NA
15. .Event capture: are the data, as collected, able to identify the intervention and outcomes if they actually occurred?	Yes	Yes	Yes	Yes	Yes	Yes	Yes	Yes
16. Disease history: is there a link between the natural history of the disease being studied and the time period for analysis?	NA	NA	NA	NA	NA	NA	NA	NA
17. Resource valuation: for studies that examine costs, have the authors defined and measured an exhaustive list of resources affected by the intervention given the perspective of the study and have resource prices been adjusted to yield a consistent valuation that reflects the opportunity cost of the resource?	NA	NA	NA	NA	NA	NA	NA	NA
18. Control variables: if the goal of the study is to examine treatment effects, what methods have been used to control for other variables that may affect the outcome of interest?	Yes	Yes	Yes	Yes	Yes	None^j^	None^k^	Yes
19. Statistical model: have the authors explained the rationale for the model/statistical method used?	No	No	No	No	No	Partial^l^	No	Yes
20. Influential cases: have the authors examined the sensitivity of the results to influential cases?	NA	NA	NA	NA	NA	NA	NA	NA
21. Relevant variables: have the authors identified all variables hypothesized to influence the outcome of interest and included all available variables in their model?	Yes	Yes	Yes	Yes	Yes	No^m^	No	No
22. Testing statistical assumptions: do the authors investigate the validity of the statistical assumptions underlying their analysis?	Yes	Yes	Yes	Yes	Yes	No	No	No
23. Multiple tests: if analyses of multiple groups are carried out, are the statistical tests adjusted to reflect this?	NA	NA	NA	NA	NA	NA	NA	NA
24. Model prediction: if the authors utilize multivariate statistical techniques in their analysis, do they discuss how well the model predicts what it is intended to predict?	No	No	No	No	No	NA	NA	Yes
25. Theoretical biases: have the authors provided a theory for the findings and have they ruled out other plausible alternative explanations for the findings?	Partial^n^	Partial^n^	Partial^n^	Partial^n^	Partial^n^	Partial^n^	No	Yes
26. Practical versus statistical significance: have the statistical findings been interpreted in terms of their clinical or economic relevance?	No	No	No	No	No	Yes	Partial^o^	Yes
27. Generalizability: have the authors discussed the populations and settings to which the results can be generalized?	Partial^p^	No	No	No	Partial^p^	No	No	Yes
**Overall level of quality**	**Very low**	**Very low**	**Very low**	**Very low**	**Very low**	**Very low**	**Very low**	**Very low**

^a^Single data source

^b^It is indicated in the text that a hypothesis was created a priori, but no more information is revealed

^c^It is not explicitly stated in the text that a hypothesis was created a priori

^d^They state that population-based studies can be generalized to the community more easily than center-specific studies because they represent the broad range of conditions under which surgeries are conducted on diverse populations in a wide range of settings by many surgeons with varying levels of experience

^e^Only inclusion criteria were described

^f^The inclusion/exclusion criteria were described only in part

^g^Only exclusion criteria were described

^h^Criteria were mentioned, but the impact on findings was not discussed

^i^No explicit procedure codes were used

^j^Only bivariate statistical analyses were conducted – no multivariate regression model was applied

^k^Only causative factors are listed descriptively

^l^Reasons for using the conducted statistical tests were given

^m^Only a low volume hospital with outpatient thyroidectomy was the object of analysis

^n^The authors did not rule out other possible interpretations

^o^Authors refer only to one paper that also tests the respective hypothesis

^p^It is stated that the current results are of limited generalizability

Moreover, the validity of the endpoints used is also questionable. None of the safety endpoints were reported in relation to surgeon or hospital volumes, only one study reported on a mortality endpoint [28], three on morbidity endpoints [22, 23, 26], one on disease specific QoL endpoint [27], and none of patient satisfaction. Day surgery related outcomes such as frequency of hospital infections or venous thrombosis embolisms were not reported in any of the studies.

External validity of the data is questionable as well. Even though the conclusions of all the included studies are based on clinical practice data, the potential patient selection bias or the retrospective study design and the questionable generalizability undermine the external validity. Generalizability of the data is put in question because all the studies (except for [27]) were conducted in the USA where the definition of day surgery and outpatient surgery may vary [5].

### Critical considerations and contradictory evidence

A critical synthesis has to be made to draw attention on the complexity of the VOR and thereof derived administrative MVSs. It is crucial to consider the over-deterministic nature of this relationship in order to avoid possible methodological drawbacks in the study design and to guarantee explanatory power [12, 13].

Without a doubt, taking into account risk-adjustment and case mix is imperative as the first step toward getting unbiased estimates of the effect of volume on outcome, but volume of surgeries can only be a proxy for higher quality. Halm et al. point out that besides general methodical shortcomings, studies investigating the VOR were not able to determine what surgical abilities or management skills of the surgery unit are enhanced by volume and why they should be uniquely related to volume [11].

Whether high-quality hospitals or surgeons are more likely to be sought by patients in the first place and are therefore capable of accumulating a higher-volume is also important to consider. Word of mouth takes a substantial part in the decision of where to undergo treatment that is often neglected in studies and health care research [10]. Also, the question whether patients of high-volume providers are more likely to be selected into treatment by the provider compared to low-volume surgeons or hospitals is mainly unanswered [11].

When conducting volume-outcome analysis, one has to be cautious not to fall in a mono-causal or reverse causality trap when establishing links between two or more variables. An observed correlation does not necessarily indicate causation. Against this backdrop, it is important to synthesize the various approaches to emphasize the complexity of the VOR and its derived policies. A scientific and surgery specific examination of the study situation is necessary to establish evidence-based minimum quantities. Quality is influenced by other factors such as the application of the best treatment methods. These factors should always supplement quality assurance focused on minimum quantities.

### Limitations

The evidence base found was only partly relevant for answering the research questions. The retrospective study design can at best show correlations between surgeon/hospital volumes and day surgery outcomes, however, its results are of limited certainty and none of the studies answered the question on the threshold MVSs.

In the systematic literature search, we only found VOR data on eight interventions, which, however, is not a representative sample of all the interventions eligible for day surgery.

Also, the consistency of definitions of the included studies is questionable. The reason is that the US’ use of the term day surgery may include 23 h stay surgery, which in the EU is seen as an inpatient surgery with a 1 day LOS.

Furthermore, the following studies on arthroscopy, meniscal repair, and colonoscopy [29–31] were excluded from the analysis even though they met the inclusion criteria. The reason was that they were found in an additional search in the end stage of the report when including them was no longer feasible. Additional reason for not including them was their assumed low marginal utility as quality of all studies was low due to their retrospectives study design.

## Conclusion

The need for a shift of surgical interventions from the inpatient setting to the day surgery setting is advocated in the international literature. The reasons for the shift include organizational, ethical, economic, and medical aspects and quality assurance in the process is argued to be key. Because the VOR does have some standing in the inpatient setting, the role of MVSs applied to the day surgery setting was scrutinized in this report. Surely, identifying possible analogies between the inpatient and the day surgery settings can serve as a valuable decision-making foundation, however, the lack of evidence of clearly established MVSs, methodical issues, and the different nature of inpatient and day surgery settings make the simple transition of inpatient results to the day surgery setting questionable.

Not only is the theory of MVSs in part questionable, but also the results from our systematic review cannot offer any clear-cut MVS thresholds. This present report, however, provides some evidence in favor of VOR, even though it based on low quality retrospective data-analyses. Two out of eight studies did not even suggest a VOR at all and so we argue that the application of MVSs should be done with caution. Moreover, because establishing the VOR and henceforth the MVSs is possible, quality prospective controlled evidence for the day surgery setting is required. Also, other quality assurance standards such as standards focusing on process and outcome quality should be taken into account.

In terms of adequate policy implications, if optimal surgery specific MVSs can be established as a quality standard and it is secured by a high-quality body of evidence for the VOR, then it is also indispensable to derive an appropriate public policy to disseminate this information to payers, health care consumers, and clinicians. Prima facie, there are three different policy approaches: (1.) utilizing the data on volumes and outcomes to enhance performance, (2.) adopting measures to limit the and (3.) publication or dissemination of the data on volume.

## Supplementary information

**Additional file 1.**

## Data Availability

Data generated or analyzed during this study are included in this published report: *Stanak, M. and Strohmaier, C. (2019): Minimum volume standards for quality assurance in day surgery: Fundamentals and Systematic Review. HTA-Projektbericht 125.*

## Abbreviations

AA: Association of Anesthetists
ACL: Anterior cruciate ligament
BADS: British Association for Day Surgery
COI: Conflict of interests
EUnetHTA: European Network for Health Technology Assessment
IAAS: International Association for Ambulatory Surgery
IQWiG: Institute for Quality and Efficiency in Healthcare
ISPOR: International Society for Pharmacoeconomics and Outcomes Research
LOS: Length of stay
GRADE: Grading of Recommendations Assessment, Development and Evaluation
MVS: Minimum volume standards
PCR: Posterior capsule rupture
REA: Rapid Relative Effectiveness Assessment
RR: Relative risk
VOR: Volume-outcome relationship
