# Supplementary material for: Minimum volume standards in day surgery: a systematic review
Source: BMC Health Serv Res. 2020 Sep 18;20:886. doi: 10.1186/s12913-020-05724-2 (PMC7501608; doi:10.1186/s12913-020-05724-2)
Supplement: Supplementary file 1 — Additional file 1. [file 12913_2020_5724_MOESM1_ESM.docx]

**Supplementary material**

**Search strategies:**

### Search strategy for Cochrane

| Search Name: Minimum Volume Standards | |
| --- | --- |
| Search Date: 12/07/2019 | |
| ID | Search |
| #1 | MeSH descriptor: [Hospitals, High-Volume] explode all trees |
| #2 | MeSH descriptor: [Hospitals, Low-Volume] explode all trees |
| #3 | (volume NEXT outcome):ti,ab,kw (Word variations have been searched) |
| #4 | ("minimum volume* standard*") (Word variations have been searched) |
| #5 | (fallzahl*) (Word variations have been searched) |
| #6 | (mindestfallzahl*) (Word variations have been searched) |
| #7 | ((surgeon* OR surgic* OR surger*) NEXT volume*):ti,ab,kw |
| #8 | #1 OR #2 OR #3 OR #4 OR #5 OR #6 OR #7 |
| #9 | MeSH descriptor: [Ambulatory Surgical Procedures] explode all trees |
| #10 | MeSH descriptor: [Outpatient Clinics, Hospital] explode all trees |
| #11 | ((ambula* OR outpatient* or day*)) (Word variations have been searched) |
| #12 | #9 OR #10 OR #11 |
| #13 | #8 AND #12 with Cochrane Library publication date Between Jan 2000 and Jul 2019 (Word variations have been searched) |
| #14 | #8 AND #12 with Publication Year from 2000 to 2019, in Trials (Word variations have been searched) |
| #15 | #13 OR #14 (Word variations have been searched) |
| Total: 62 Hits | |

### Search strategy for CRD

| Search Name: Minimum Volume Standards | |
| --- | --- |
| Search Date: 12/07/2019 | |
| ID | Search |
| #1 | (minim* volume* standard*) |
| #2 | (volume-outcome) |
| #3 | ((surgeon* OR surgic* OR surger* OR hospital* OR procedur*) NEXT (volume* OR case-load* OR caseload*)) |
| #4 | #1 OR #2 OR #3 |
| #5 | (ambula* OR outpatient* OR day*) |
| #6 | MeSH DESCRIPTOR Ambulatory Surgical Procedures EXPLODE ALL TREES |
| #7 | MeSH DESCRIPTOR Outpatient Clinics, Hospital EXPLODE ALL TREES |
| #8 | MeSH DESCRIPTOR Outpatients EXPLODE ALL TREES |
| #9 | #5 OR #6 OR #7 OR #8 |
| #10 | #4 AND #9 |
| #11 | (#10) FROM 2000 TO 2019 |
| Total: 24 Hits | |

### Search strategy for Medline

| Search Name: Minimum Volume Standards | |
| --- | --- |
| Search Date: 12/07/2019 | |
| ID | Search |
| #1 | exp *Hospitals, High-Volume/(1087) |
| #2 | exp *Hospitals, Low-Volume/(654) |
| #3 | (volume adj outcome).ti,ab. (806) |
| #4 | ((surgeon* or surgic* or surger* or hospital* or procedur* or case or minim*) adj (volume* or case-load* or caseload*)).ti,ab. (8048) |
| #5 | 1 or 2 or 3 or 4 (8866) |
| #6 | exp Ambulatory Surgical Procedures/(12921) |
| #7 | exp Outpatient Clinics, Hospital/(17805) |
| #8 | surgery.fs. (2161180) |
| #9 | 9 and 10 (397) |
| #10 | ((ambulatory* or outpatient* or day*) adj3 (surge* or surgic* or procedure*)).ti,ab. (67012) |
| #11 | 8 or 11 or 12 (72907) |
| #12 | 7 and 13 (356) |
| #13 | minim* volume* standard*.mp. (42) |
| #14 | 14 or 15 (398) |
| #15 | remove duplicates from 16 (314) |
| #16 | limit 17 to yr="2000 - 2019" (288) |
| #17 | exp *Hospitals, High-Volume/(1087) |
| #18 | exp *Hospitals, Low-Volume/(654) |
| Total: 288 hits | |

### Search strategy for Embase

| Search Name: Minimum Volume Standards | |
| --- | --- |
| Search Date: 12/07/2019 | |
| ID | Search |
| #1 | 'surgical volume'/exp |
| #2 | 'hospital volume'/exp |
| #3 | 'high volume hospital'/exp |
| #4 | 'low volume hospital'/exp |
| #5 | (volume* NEAR/1 outcome*):ti,ab |
| #6 | ((surgeon* OR surgic* OR surger* OR hospital* OR procedur* OR case OR minim*) NEAR/1 (volume* OR 'case load*' OR caseload*)):ti,ab |
| #7 | #1 OR #2 OR #3 OR #4 OR #5 OR #6 |
| #8 | 'ambulatory surgery'/exp |
| #9 | ((ambulatory* OR outpatient* OR day*) NEAR/2 (surge* OR surgic* OR procedure*)):ti,ab |
| #10 | #8 OR #9 |
| #11 | #7 AND #10 |
| #12 | 'minim* volume* standard*':ti,ab,de |
| #13 | fallzahl* |
| #14 | mindestfallzahl* |
| #15 | frequenzregel* |
| #16 | #12 OR #13 OR #14 OR #15 |
| #17 | #11 OR #16 |
| #18 | (#11 OR #16) AND [2000-2019]/py |
| #19 | #18 AND 'conference abstract'/it |
| #20 | #18 NOT #19 |
| Total: 346 hits | |

### Search strategy for Livivo

**Date:** 12/07/2019

Freie Suche: (((mindestfallzahl* OR mindestmeng* OR fallzahl* OR frequenzregel*) AND (chirurg* OR operat* OR eingriff* OR OP)) AND (Ambula* OR Tages*))

Publikationsdatum eingeschränkt auf: 2000-2019

**Total:** 33 hits
